# Waldenström Macroglobulinemia: The Role of TP53 Mutations in Disease Progression and Therapeutic Response

**DOI:** 10.3390/cimb47040260

**Published:** 2025-04-08

**Authors:** Despoina Dimitria Kampitsi, Paschalis Theotokis, Paschalis Evangelidis, Soultana Meditskou, Maria Eleni Manthou, Iasonas Dermitzakis

**Affiliations:** 1 Department of Histology-Embryology, School of Medicine, Aristotle University of Thessaloniki, 54124 Thessaloniki, Greece; dkampitsi@auth.gr (D.D.K.); ptheotokis@auth.gr (P.T.); sefthym@auth.gr (S.M.); mmanthou@auth.gr (M.E.M.); 2 Hematology Unit-Hemophilia Centre, 2nd Propedeutic Department of Internal Medicine, Hippocration Hospital, Aristotle University of Thessaloniki, 54642 Thessaloniki, Greece; pascevan@auth.gr

**Keywords:** prognosis, TP53, treatment, Waldenström Macroglobulinemia

## Abstract

Waldenström Macroglobulinemia (WM) is a rare, indolent B-cell lymphoproliferative disorder characterized by the production of monoclonal IgM paraprotein and infiltration of the bone marrow by lymphoplasmacytic cells. While WM generally exhibits a slow clinical course, it has the potential to progress into more aggressive hematologic malignancies, such as diffuse large B-cell lymphoma. The *TP53* gene, often referred to as the “guardian of the genome”, plays a pivotal role in maintaining genomic stability, regulating the cell cycle, and orchestrating apoptosis. Mutations in *TP53* undermine these essential processes, resulting in dysregulated cellular proliferation, defective apoptotic mechanisms, and genomic instability—hallmarks of cancer development. Although *TP53* mutations have been extensively investigated in several hematologic malignancies, including acute myeloid leukemia, myelodysplastic syndromes, and chronic lymphocytic leukemia, their role in WM remains underexplored. Emerging evidence suggests that *TP53* mutations may have a significant impact on the disease progression and therapeutic response in WM. This review examines the current knowledge of *TP53* mutations in WM, highlighting their implications for prognosis and therapeutic strategies. A deeper understanding of the role of *TP53* in WM could provide critical insights for improving disease management and advancing the development of targeted therapies.

## 1. Introduction

Waldenström Macroglobulinemia (WM) is a rare, indolent form of non-Hodgkin lymphoma characterized by the infiltration of bone marrow with monoclonal immunoglobulin M (IgM)-secreting lymphoplasmacytic lymphoma (LPL) cells [1]. WM accounts for approximately 1% to 2% of all hematologic malignancies and is almost invariably preceded by IgM monoclonal gammopathy of undetermined significance (MGUS) [2,3]. The disease predominantly affects males, with a median age of onset of 63 years [2]. At the time of diagnosis, approximately 75% of patients present with symptoms, most commonly due to anemia resulting from bone marrow infiltration by LPL cells [4]. Hyperviscosity syndrome, caused by excessive accumulation of monoclonal IgM in the blood, occurs in 10% to 30% of cases. This condition can be life-threatening and necessitates urgent medical intervention. While WM generally arises sporadically, approximately 20% of affected individuals have a first-degree relative to WM or another B-cell lymphoma, suggesting a potential genetic predisposition [5,6]. Moreover, patients with WM are at an increased risk of developing other malignancies, including both hematologic and solid cancers [7]. Currently, there is no curative treatment for WM [4]. The disease follows a relapsing course, and 2% to 10% of cases may progress to aggressive non-Hodgkin lymphoma, such as diffuse large B-cell lymphoma (DLBCL) [8,9,10,11]. The median overall survival (OS) for patients with WM is approximately five years [12,13].

While WM remains an incurable disease, advancements in molecular genetics have provided critical insights into its pathogenesis. Several mutations and genetic alterations have been implicated in WM. The *MYD88* L265P mutation, present in 93–97% of cases, and the *CXCR4* WHIM mutation, detected in up to 40%, are recognized as key genetic hallmarks of the disease [14,15,16,17,18,19,20]. Additionally, 6q deletions, observed in up to 50% of cases, suggest an independent pathway of disease progression by affecting genes involved in NF-κB signaling, apoptosis, and B-cell survival [21,22]. *ARID1A* mutations, occurring in 17% of cases, disrupt chromatin remodeling and tumor suppression [19]. Furthermore, *CD79A* and *CD79B* mutations, identified in 8–12% of cases, enhance B-cell receptor signaling, frequently in conjunction with *MYD88* mutations, potentially contributing to disease progression and transformation [19,23,24]. Trisomy 4 and 13q14 deletion have also been reported [25,26]. Although these genetic alterations play a significant role in tumorigenesis, the impact of *TP53* in WM remains incompletely understood. TP53 is a crucial regulator of genomic stability, governing cell cycle checkpoints, DNA repair, and apoptosis. In various hematologic malignancies, *TP53* aberrations are associated with aggressive disease phenotypes and poor prognosis [27]. Although *TP53* mutations are less frequent in WM compared to other B-cell lymphomas, emerging evidence suggests that their presence may contribute to disease progression and therapeutic response, particularly in cases that undergo transformation into DLBCL [28,29,30,31].

Despite extensive research on the molecular landscape of WM, the precise role of *TP53* in the disease remains inadequately understood. Its potential as a prognostic marker and therapeutic target warrants further investigation. This narrative review aims to provide a focused discussion on the significance of *TP53* in WM, summarizing current knowledge on its molecular function, its impact on disease evolution, and the implications for future treatment strategies. By highlighting the clinical relevance of *TP53* dysfunction, this review seeks to contribute to a deeper understanding of the disease evolution and inform potential therapeutic approaches.

## 2. Prevalence and Genetic Landscape of *TP53* Mutations in WM

*TP53* abnormalities could manifest in various genetic alterations, including mutations within the *TP53* gene and chromosomal changes, such as deletion of the short arm of chromosome 17. This deletion is significant as it encompasses a minimally deleted region on chromosome 17p, which contains 79 genes, consistently leading to the loss of *TP53* [32]. Another notable genetic alteration is acquired uniparental disomy, a somatic event where a genomic region loses its biparental inheritance, resulting in two identical copies from a single parent [32,33]. This phenomenon leads to loss of heterozygosity without a deletion, potentially making a recessive mutation or oncogene amplification dominant. The frequency of *TP53* mutations in WM patients varies widely across studies, ranging from 2.3% to 29% [23,27,28,29,31,32,33,34,35,36,37,38,39,40]. Large-scale studies reported mutation rates of 2.3%, 4.9%, 8%, and 24.8% in cohorts of 265, 239, and 210 WM patients, respectively [29,33,39]. Importantly, no correlation has been observed between *TP53* mutations and patient age [40]. This variation arises from the application of different techniques, each exhibiting varying degrees of sensitivity.

The *TP53* gene exhibits substantial genetic variability, predominantly characterized by missense mutations, while frameshift alterations are significantly less frequent, and nonsense mutations are only rarely documented [32,34]. The majority of *TP53* mutations are localized within the DNA-binding domain, which spans exons 5 through 8 and is critical for the protein’s function [32,33,34,35,41]. In MWCL-1, a recently characterized WM cell line, the resultant p53 protein is designated as p53^V143A^ [41]. The functionality of this mutant TP53 variant has been evaluated by examining the activity of TP53 and its downstream targets, including p21, PUMA, and MDM2, in response to treatment with nutlin-3a [32]. Given that cells harboring *TP53* mutations typically exhibit resistance to nutlin-3a, this investigation aimed to determine whether p53^V143A^ retains any functional capacity. Nutlin-3a is a small-molecule inhibitor that stabilizes TP53 by preventing its interaction with MDM2, a negative regulator that promotes TP53 degradation. In *TP53*-mutant cells, p53 activation was not observed despite nutlin-3a treatment, indicating that the mutation renders TP53 non-functional. The p53^V143A^ variant specifically lacks transcriptional activity at physiological temperature (37 °C) [41]. Interestingly, at subphysiological temperatures, p53^V143A^ can induce the expression of certain genes involved in cell cycle arrest and DNA repair, although it remains incapable of triggering apoptosis. Under specific conditions, p53^V143A^ may partially regain functionality. This finding has significant implications for cancer therapeutics, as it may inform novel strategies to stabilize or reactivate mutant TP53 proteins for therapeutic benefit [42].

Mutations in the *TP53* gene in patients with WM are hypothesized to be associated with genomic instability and the acquisition of additional co-occurring mutations. However, findings across studies have been inconsistent. Some investigations have reported no significant association between *TP53* mutations and chromothripsis or other genetic alterations, including mutations in *MYD88*, *CD79A*/*CD79B*, *CXCR4*, *BTK*, or *IGHV* [32,35,40]. Conversely, other studies suggest that *TP53* mutations frequently co-occur with *MYD88* mutations, particularly the *MYD88* L265P variant and homozygous *MYD88* mutations [33,35]. Additionally, *TP53* mutations have been commonly identified in conjunction with *CXCR4* mutations [29,33,35,39]. The temporal relationship between *TP53* and *CXCR4* mutations remains unclear, and it has yet to be determined whether *TP53* mutations specifically arise within *CXCR4*-mutated cells. This uncertainty underscores the necessity of continuous monitoring in patients with *CXCR4* mutations to detect the emergence of additional high-risk genetic aberrations. The distribution of *TP53* abnormalities across complex karyotype subgroups is not uniform. They are more frequently observed in cases with complex karyotypes that lack trisomies [33]. Furthermore, *TP53* mutations have been associated with an increased frequency of copy number alterations compared to patients harboring wild-type *TP53*, particularly deletions affecting 6q, 7q, and 17p [29,32,33]. In certain cases where both 17p deletions and *TP53* mutations are present, the underlying mechanism appears to involve acquired uniparental disomy. The MWCL-1 cell line also exhibits an association between *TP53* mutations and 17p deletion [41]. However, some studies have failed to confirm this correlation, potentially due to the limited sensitivity of sequencing technologies and the low proportion of cells harboring 17p deletions within the analyzed samples [27,33,43].

Only a limited number of recurrent chromosomal abnormalities have been identified in WM, partly due to the difficulty in obtaining tumor metaphases for karyotypic analysis [43]. To circumvent this challenge, alternative cytogenetic techniques, such as fluorescence in situ hybridization (FISH), have been utilized to investigate the disease. Of particular interest, interstitial deletions can result in the loss of a single copy of *TP53* without altering the overall chromosome 17 copy number, as evidenced by the retention of two signals from the centromeric probe in FISH analyses [26]. The reported frequency of 17p deletions varies across studies, with prevalence rates of approximately 15%, 8%, and 7% in cohorts of 40, 140, and 239 WM patients, respectively, as determined using FISH and/or CBA [26,29,43]. Deletion of 17p, which encompasses *TP53*, has been observed with a higher prevalence in female patients, occurring in 5 out of 16 women compared to only 1 out of 24 men in one cohort [26]. Additionally, patients with chromosomal translocations exhibit a significantly increased likelihood of harboring the 17p deletion, an association that has been found to be statistically significant [43].

In this context, *TP53* abnormalities are not considered to play a primary role in the etiology of WM [26]. At the time of diagnosis, *TP53* aberrations may be present in only a small subset of malignant cells. This notion is supported by the detection of *TP53* mutations in 3 out of 57 (5%) patients with IgM MGUS [23]. Similarly, a separate study reported no detectable *TP53* mutations in a cohort of 10 MGUS patients [32]. The presence of *TP53* abnormalities at disease onset appears to be infrequent, as *TP53* mutations are more commonly observed in later stages of the disease. Studies indicate that 80% of patients with disease progression harbor *TP53* mutations compared to only 24.8% of patients across all disease stages [39]. Furthermore, two independent studies found that *TP53* mutations or 17p deletions were detected at diagnosis in only one out of four and one out of six patients, respectively, with the remaining cases developing these abnormalities as the disease advanced or following treatment [26,40]. These findings suggest that *TP53* abnormalities arise as a consequence of genomic instability rather than being pre-existing drivers of the disease. Notably, *TP53* abnormalities are typically present in the majority of malignant cells, implying that the affected clones possess a selective survival or proliferative advantage, thereby contributing to their expansion and evolution over the course of the disease [26,32].

## 3. The Role of *TP53* in the Prognosis of WM

While WM is generally considered an indolent B-cell malignancy, the presence of *TP53* abnormalities is associated with more aggressive disease behavior, poorer OS, and shorter progression-free survival (PFS). Understanding the impact of *TP53* abnormalities on prognosis is essential for refining risk stratification and optimizing therapeutic strategies for patients with WM.

Several studies have failed to establish a statistically significant correlation between TP53 gene mutations or deletions and OS, indicating no substantial difference in survival outcomes based on the presence or absence of TP53 abnormalities [23,26,40,44]. One study reported that patients with 17p deletions exhibited a higher likelihood of mortality at the last follow-up compared to those without the deletion [26]. However, definitive conclusions remain elusive due to several confounding factors, including patient heterogeneity, differences in treatment regimens, the limited number of events, and the variability in the timing of chromosomal abnormality detection (at diagnosis vs. later in the disease course). Moreover, another study emphasized the necessity for long-term investigations to validate these findings, as the prognostic impact of 17p deletions may become more pronounced over an extended timeframe [44]. The median follow-up duration in the study was approximately 39.7 months, which may be insufficient to fully capture the long-term effects of 17p deletion on survival outcomes. Although, in patients undergoing BTKi therapy, a positive TP53 status was strongly linked to disease progression, multivariate analysis indicated a similar trend toward significance, but the limited cohort size prevented it from reaching statistical significance [40].

Mutations in the *TP53* gene have been shown to exert a negative impact on OS in several other studies (Table 1) [34]. In univariate analysis, *TP53* abnormalities were significantly associated with reduced OS, and in multivariate analyses, they remained one of the only independent prognostic factors alongside the International Prognostic Scoring System for WM [29]. Specifically, among patients with *TP53* abnormalities WM, the median OS for symptomatic WM was 4 years, while for indolent WM, it was 9 years, both of which were significantly shorter compared to WM cases without *TP53* alterations [32]. Similarly, patients with *TP53* mutations demonstrated a markedly reduced OS, with a median survival of 53.5 months [38]. Patients harboring *TP53* mutations more frequently present with treatment-requiring or symptomatic disease [34]. Specifically, 91.7% of patients with a *TP53* mutation received treatment compared to 54.7% of TP53 wild-type patients. *TP53* deletions are more likely to have undergone prior therapy before enrolling in clinical trials [26]. Additionally, a shorter time to treatment (TTT) has been observed in patients with *TP53* abnormalities, with a median TTT of 2 years, in contrast to 5 years in TP53WT patients [32]. However, some studies have not identified a statistically significant association between *TP53* mutations and shorter TTT, suggesting that additional factors may contribute to treatment initiation in these patients [23].

Furthermore, *TP53* abnormalities have been identified as a significant prognostic factor for shorter PFS in patients with WM [29]. In multivariate analysis, *TP53* abnormalities remained the sole factor with a significant negative impact on PFS. Notably, patients with 17p deletions exhibited a median PFS of 18.7 months compared to 30 months in those without, demonstrating a statistically significant difference [43]. Another study further reinforced this association, revealing that *TP53* mutations correlate with poorer PFS in WM patients, including those who received treatment [34]. However, some studies, particularly those with smaller patient cohorts, have reported no predictive value of *TP53* mutations in relation to PFS [34,36]. Regarding time to progression (TTP), patients with *TP53* abnormalities experienced a significantly shorter TTP, with a median of 18 months compared to 48 months in *TP53* wild-type patients [32]. Additionally, several clinical features indicative of an aggressive disease course appear to be linked to 17p deletions. Specifically, two studies have confirmed a greater extent of bone marrow involvement in patients with 17p deletions, further supporting the association between 17p deletions and more advanced disease [26,43]. In the context of WM transformation into DLBCL, recurrent acquired *TP53* abnormalities have been identified in DLBCL patients, underscoring the role of *TP53* in disease progression [28,29,30,31]. Notably, 87% (6 out of 7) of patients who underwent transformation to DLBCL harbored *TP53* abnormalities. Furthermore, another study reported that both patients (2/2) with *TP53* mutations progressed to DLBCL, whereas none of the 43 patients with *TP53* wild-type experienced transformation [29,31]. Although the current literature does not pinpoint a specific molecular pathway, we propose that future research should focus on elucidating how TP53 mutations mechanistically drive WM progression and transformation into DLBCL, particularly through its roles in cell cycle regulation, DNA damage repair, and apoptosis, which may offer insights into potential therapeutic targets [31]. One additional area for future research is the uncertainty regarding whether, in all cases, the progression to DLBCL results from the transformation of the malignant clone(s), or whether it represents a de novo DLBCL that is clonally distinct from WM [30].

## 4. Evaluating the Influence of TP53 on WM Therapy

In WM, alterations in *TP53*, including mutations and deletions, have been linked to treatment resistance and inferior clinical outcomes. Patients harboring *TP53* mutations often exhibit reduced sensitivity to standard therapies, including chemoimmunotherapy and novel targeted agents, leading to higher relapse rates and shorter response durations. Given its impact on therapeutic efficacy, the presence of *TP53* mutations has emerged as a critical biomarker for treatment decision-making in WM, necessitating the exploration of alternative therapeutic approaches tailored to this high-risk subgroup.

G-1 and its enantiomer LNS8801 are potent activators of G-protein-coupled estrogen receptor 1 (GPER1), a membrane-bound receptor that induces a significant upregulation of *TP53* expression [45]. In vitro studies have demonstrated that G-1 strongly induces the expression of TP53 and its key downstream targets, including p21, BAX, BAD, and PUMA, in CD19^+^ cells derived from WM patients. Furthermore, genetic silencing of *TP53* markedly attenuated the growth-inhibitory effects of G-1, underscoring the pivotal role of *TP53* in mediating its antitumor activity. *TP53* mutations significantly impair the normal function of TP53, a critical tumor suppressor responsible for eliminating malignant cells [32]. When *TP53* is mutated, cancer cells exhibit increased resistance and survival, even in the presence of treatments designed to eradicate them. This finding has profound clinical implications, suggesting that patients harboring *TP53* mutations may demonstrate poor responsiveness to therapies targeting the TP53 pathway. As a result, the development of novel therapeutic strategies capable of overcoming the functional loss of TP53 associated with TP53 mutations is imperative. Given that impaired *TP53* function is a well-established driver of chemoresistance, effective drug development strategies targeting tumors with mutant *TP53* remain a significant challenge. Various compounds have been evaluated to either bypass the defective TP53 pathway or restore TP53 activity in mutant-expressing cells; however, their translation into successful clinical therapies has been inconsistent. Addressing these limitations requires a deeper understanding of *TP53*-dependent mechanisms and the development of targeted interventions tailored for *TP53*-mutated malignancies.

PrimaMet (APR-246) and CP-31398 have been proposed to bind to mutant TP53 proteins by interacting with the DNA-binding domain, promoting proper protein folding, and restoring p53 function [32]. Treatment with PrimaMet (APR-246), CP-31398, and ibrutinib has demonstrated significant therapeutic efficacy. However, in vitro studies have shown no induction of *TP53* expression or activation of its downstream targets, including p21, MDM2, and PUMA, suggesting that their cytotoxic effects are independent of the TP53 pathway. Notably, alterations in the *TP53* gene do not appear to affect the response of WM patients to ibrutinib, as those harboring *TP53* mutations exhibited a significant therapeutic response [32,33]. Consistent with these findings, a Western blot analysis revealed no significant changes in TP53 levels following PRIMA-1Met treatment [46]. Moreover, selectively knocking down TP53 did not affect PRIMA-1Met-induced apoptosis in WM cells, suggesting a potentially *TP53*-independent mechanism. Interestingly, PRIMA-1Met treatment led to the activation of p73, a member of the TP53 superfamily that shares both structural and functional similarities with TP53. Taken together, these data indicate that PrimaMet, CP-31398, and ibrutinib represent viable therapeutic options for WM patients harboring TP53 mutations (Table 2). While both preclinical and clinical data show that ibrutinib exhibits activity in TP53-mutated WM patients, further investigation is necessary to identify novel treatments for this high-risk group. A phase II trial assessing ibrutinib in previously untreated WM patients, incorporating serial whole-exome sequencing, has now been completed. The results of this trial will offer valuable insights into the mechanisms of ibrutinib resistance, as well as its impact on clonal evolution (ClinicalTrials.gov Identifier: NCT02604511) [35].

Furthermore, the Phase 3 ASPEN trial compared the efficacy of zanubrutinib and ibrutinib in a cohort of 190 patients, including both treatment-naïve and relapsed cases, with 24.8% harboring *TP53* mutations [39]. Among these, 98 patients received zanubrutinib, while 92 were treated with ibrutinib. This study reported that TP53 mutations negatively affected the maximum response rate (MRR) and PFS in patients treated with ibrutinib, but not in those treated with zanubrutinib. This suggests that more potent BTKis, like zanubrutinib, may enhance the response in WM patients with TP53 mutations. Consistently, TP53 mutations were associated with refractory disease and diminished OS following the development of resistance to ibrutinib [35]. Notably, patients harboring TP53 mutations demonstrated no response to salvage therapy, despite prior exposure to four distinct treatment classes. Similarly, in those receiving a fixed-duration, chemotherapy-free regimen, TP53 mutations correlated with significantly reduced OS [38]. Conversely, an analysis of symptomatic relapsed/refractory WM patients undergoing combination therapy with idelalisib and obinutuzumab for a fixed two-year duration revealed no evidence supporting the predictive value of TP53 mutations in treatment response [36].

The necessity of rituximab maintenance therapy in WM remains a topic of ongoing debate. However, patients—including those harboring prognostically adverse *TP53* mutations—who receive rituximab maintenance therapy, either as monotherapy or in combination, exhibit a significant extension in both PFS and OS [34]. Notably, in patients previously treated for lymphoma, the presence of clonal hematopoiesis of indeterminate potential (CHIP) has been associated with an increased risk of therapy-related myeloid neoplasms (TMNs) following autologous stem cell transplantation [47]. Future studies assessing the risk of TMNs following cytotoxic chemotherapy in WM patients with CHIP would provide valuable insights, aiding in the identification of individuals at higher risk who may require alternative treatment approaches [33]. Additionally, WM patients with a 17p deletion may benefit from tailored therapeutic strategies similar to those employed in chronic lymphocytic leukemia [43]. The prognostic relevance of 17p deletion should be further validated in patients undergoing novel treatment combinations, particularly those incorporating monoclonal antibodies. Regarding the use of frontline dexamethasone, evidence suggests a significantly shorter OS and a potentially shorter PFS in patients with *TP53* mutations [34]. However, when multivariate analysis incorporating all genetic and treatment factors was performed, the statistical significance of this effect was diminished. This may be attributed to the limited number of cases with complete genetic and treatment data, suggesting that the observed negative impact of first-line dexamethasone was not directly dependent on *TP53* status. For WM/LPL patients harboring *TP53* mutations, evidence suggests that ibrutinib, rather than a dexamethasone-based regimen, represents a more appropriate first-line therapeutic option.

TP53 mutation testing is not currently included in either the IPSSWM or the Mayo Stratification of Risk system for WM. According to current Reports of Consensus Panel from International workshops on WM, for patients initiating therapy and in cases of aggressive relapse with rapidly worsening refractoriness, it is essential to test for TP53 [48,49,50]. TP53 abnormalities are important for prognosis and adjusting treatment plans, as these mutations may not be present at diagnosis but could develop later during disease progression, especially in relapsing or refractory patients. Testing for TP53 mutations should be conducted when starting therapy and repeated before each new treatment regimen. Although the optimal frequency of molecular testing is uncertain due to limited data, understanding TP53 alterations could lead to more personalized treatment approaches. However, at present, TP53 status cannot definitively guide therapy choices.

The assessment of TP53 gene mutations should at a minimum involve Sanger sequencing of genomic DNA extracted from CD19^+^ selected bone marrow cells [50]. Since TP53 mutations may be confined to specific clonal subpopulations, the use of an NGS panel is recommended for a more comprehensive detection. Moreover, NGS can be performed on unsorted samples, making it a more practical option for clinical laboratories, as it avoids the time-intensive and costly process of cell sorting [33]. Additionally, TP53 variants have been identified in cell-free DNA (cfDNA) isolated from plasma samples [40]. The successful detection of these variants in cfDNA through targeted NGS underscores the potential of liquid biopsies as a useful tool for monitoring TP53 mutations in patients. This method enables regular disease monitoring via a simple blood test, significantly reducing the need for repeated invasive bone marrow procedures.

Based on the available data, the panel recommends performing FISH studies to assess del17p (TP53), using appropriate probes on bone marrow (BM) samples following CD19^+^ cell enrichment [50]. While alternative methods, such as SNP arrays or whole-genome sequencing, can be used in CD19^+^-enriched samples, they are not considered suitable for routine laboratory practice. The panel also noted that CD19 sorting is costly and time-consuming, which limits its widespread adoption in laboratories. However, it is still recommended due to its potential to reduce the detection of gene mutations associated with CHIP, which is particularly relevant for TP53 mutations. These mutations are common in elderly populations and hematological malignancies like WM. By enriching cells for CD19^+^, the likelihood of detecting abnormalities predominantly found in myeloid cells would be minimized.

Genetic stratification should be incorporated into future clinical trials, with highly sensitive, standardized methods used to accurately genotype participants for baseline mutations in TP53 [49]. Growing evidence suggests that TP53 abnormalities are associated with poor responses to chemoimmunotherapy in chronic lymphocytic leukemia and, to a lesser extent, to novel treatments in WM. Further research into the role of TP53 mutations is needed to fully understand their potential impact on treatment strategies. Therefore, TP53 testing and reporting are considered crucial for future clinical trials, though current knowledge is insufficient for patient stratification based on TP53 mutations alone. Considering the potential of personalized therapeutic strategies, future research could focus on developing options to correct TP53 dysfunction or target related pathways, which may offer promising therapeutic avenues associated with TP53 mutations.

## 5. Conclusions

WM is a biologically heterogeneous disease characterized by a complex genetic landscape that influences its progression and therapeutic response. Among the various molecular alterations, *TP53* mutations have emerged as a critical factor, particularly in advanced disease stages. While *TP53* mutations do not appear to play a primary role in the pathogenesis of WM, their increased prevalence in later disease phases, particularly in cases exhibiting genetic instability, underscores their significance in clonal evolution and disease progression. The presence of *TP53* abnormalities has been associated with adverse clinical outcomes, including treatment resistance and transformation to aggressive lymphoma. Despite these insights, inconsistencies across studies highlight the need for standardized methodologies and more comprehensive investigations. The integration of *TP53* testing into clinical practice is imperative for risk stratification and personalized treatment planning. Evaluating *TP53* status at diagnosis and prior to each therapeutic intervention will facilitate the identification of high-risk patients and enable tailored therapeutic approaches to improve clinical outcomes. Future research should focus on delineating the precise mechanisms through which *TP53* mutations contribute to WM progression and therapeutic resistance. Longitudinal studies incorporating single-cell sequencing and functional assays will be instrumental in uncovering the clonal dynamics of TP53-mutated subpopulations and their impact on disease evolution. Additionally, the development of innovative therapeutic strategies aimed at restoring or bypassing *TP53* function represents a promising avenue for improving treatment outcomes. As the treatment landscape for WM continues to evolve, integrating molecular profiling into routine clinical decision-making will be crucial for optimizing patient care and advancing precision medicine in this challenging disease.

## Figures and Tables

**Table 1:** Prognostic effects of TP53 abnormalities in Waldenström’s macroglobulinemia (WM).

TP53 Abnormalities	Participants	Prognosis	Effects of TP53 Abnormalities	Ref.
17p deletion	Total (n = 140); Deletion (n = 11)	Poor	Higher median percentage of tumor cells in bone marrow of patients with a 17p deletion; shorter PFS and shorter disease-free survival in patients with 17p deletion.	[43]
17p deletion	Total (n = 40); Deletion (n = 6)	Insufficient	No clear difference in OS; higher percentage of bone marrow involvement in patients with 17p deletion; greater risk for patients with 17p deletions to have passed away at the last follow-up; higher likelihood of prior treatment in patients with 17p deletion; no definitive conclusions can be drawn.	[26]
*TP53* mutation	Total (n = 62); Mutation (n = 6)	Unaltered	No statistically significant correlation between *TP53* mutations and overall survival (OS).	[23]
*TP53* mutation	Total (n = 49); Mutation (n = 11)	Unaltered	No prognostic factors for OS emerged, except for a harmful effect of the *TP53* mutation. No statistical significance in the Cox model; univariate analysis of molecular screening revealed no significant impact of *TP53* genotypes on progression-free survival (PFS).	[36]
*TP53* mutation	Total (n = 48); Mutation (n = 11)	Poor	Patients with *TP53* mutations showed lower OS. Five-year OS reached 45.5% for *TP53*-mutated patients compared to 82.3% for those without the mutation.	[38]
*TP53* mutation	Total (n = 68); Mutation (n = 11.8%)	Poor; Unaltered	Significantly worse OS and PFS rates in patients with *TP53* mutation; shorter time to treatment (TTT) and more severe clinical presentation in patients with the *TP53* mutation; no significant prognostic differences between patients with and without 17p deletion.	[34]
*TP53* mutation	Total (n = 265); Mutation (n = 6)	Poor	At the time of *TP53* mutation detection, the median bone marrow involvement was 80%, and the median hemoglobin level measured 92 g/L; the median serum Immunoglobulin M level was 25.08 g/L, with two patients exhibiting symptomatic hyperviscosity; after a median follow-up of 18 months, two patients (33%) had succumbed to progressive disease, both of whom presented with biallelic *TP53* inactivation.	[33]
*TP53* mutation	Total (n = 18); Mutation (n = 2)	Poor	Genomic mutations detected in transformed patients comprised *TP53*; all detected mutations appeared in Diffuse Large B-Cell Lymphoma (DLBCL) and contributed to NF-κB-driven lymphomagenesis.	[28]
*TP53* mutation	Total (n = 8); Mutation (n = 2)	Poor	In all cases of clonally related lymphoplasmacytic lymphoma/WM and DLBCL included in the targeted mutation analysis, transformation was marked by acquired *TP53* mutations.	[30]
*TP53* mutation	Total (n = 45); Mutation (n = 4%)	Poor	Two patients carried a *TP53* mutation, both with clonally related histological transformation to DLBCL; the remaining 43 patients with *TP53* wild-type showed no clinical or histological transformation.	[31]
*TP53* mutation	Total (n = 2); Mutation (n = 1)	Poor	Mutations in *TP53* identified in the case of early death (male, 68 years old).	[44]
*TP53* mutation	Total (n = 14); Mutation (n = 4)	Insufficient	In patients undergoing BTKi therapy, a positive *TP53* status was strongly linked to disease progression; multivariate analysis indicated a similar trend toward significance, but the limited cohort size prevented it from reaching statistical significance.	[40]
*TP53* mutation; 17p deletion	Total (n = 125); Mutation (n = 9); Deletion (n = 12)	Poor	Lower OS in the group harboring *TP53* abnormalities; higher proportion of β2-microglobulin levels and greater International Prognostic Scoring System for WM (IPSSWM) score in patients with *TP53* abnormalities; shorter median TTT in the group with *TP53* abnormalities.	[32]
*TP53* mutation; 17p deletion	Total (n = 170); Abnormalities (n = 26)	Poor	OS and PFS showed negative impact due to *TP53* abnormalities. Multivariate analyses identified *TP53* abnormalities as a significant negative factor for PFS, while both IPSSWM and *TP53* abnormalities retained significant negative effects on OS.	[29]

BTKi: Bruton’s Tyrosine Kinase Inhibitor. DLBCL: Diffuse Large B-Cell Lymphoma. IPSSWM: International Prognostic Scoring System for Waldenström Macroglobulinemia. NF-κB: Nuclear Factor kappa-light-chain-enhancer of activated B cells. OS: Overall Survival. PFS: Progression-Free Survival. TTT: Time To Treatment. Ref.: References. TP53: Tumor Protein 53. WM: Waldenström Macroglobulinemia.

**Table 2:** Impact of TP53 abnormalities on treatment outcomes in Waldenström’s macroglobulinemia (WM).

Abnormalities	Participants	Treatment Studied	Outcome	Effects of *TP53* Abnormalities	Ref.
*TP53* deletion	Total (n = 140); Deletion (n = 11)	Oral chlorambucil; Oral fludarabine	No impact	No significant interaction was observed between the impact of 17p deletion and the treatment group.	[43]
*TP53* mutation	Total (n = 7); Mutation (n = 7)	Bortezomib; Dexamethasone; Rituximab; Ibrutinib; Ixazomib; Dexamethasone; Rituximab; Bendamustine; RituximabV; Venetoclax	Impact	*TP53* mutations appear to influence the treatment outcomes of WM; WM patients harboring *TP53* mutations have demonstrated a response to ibrutinib therapy.	[33]
*TP53* mutation	Total (n = 49); Mutation (n = 11)	Chemo-free regimen	Impact	Treatment with a fixed-duration, chemo-free regimen resulted in a significantly shorter overall survival (OS) for those with *TP53* mutations.	[38]
*TP53* mutation	Total (n = 68); Mutation (n = 11.8%)	Ibrutinib; Dexamethasone; Rituximab	Impact	Ibrutinib treatment showed a trend toward improved progression free survival (PFS) after treatment among patients with *TP53* mutations; frontline dexamethasone has been linked to significantly shorter OS and potentially shorter PFS in patients with *TP53* mutations. However, when a multivariate analysis was performed, incorporating all genetic and treatment factors, the significance of this effect disappeared; maintenance rituximab was linked to significantly improved PFS in *TP53* mutated patients.	[34]
*TP53* mutation	Total (n = 20); Mutation (n = 3)	Ibrutinib; Salvage therapy	Impact	*TP53* mutations were associated with refractory disease and reduced OS after developing resistance to ibrutinib; no response to salvage therapy was observed in patients with a *TP53* mutation.	[35]
*TP53* mutation	Total (n = 190); Mutation (n = 24.8%)	Ibrutinib; Zanubrutinib	Impact	*TP53* mutations negatively affected the maximum response rate and PFS in patients treated with ibrutinib, but not in those treated with Zanubrutinib.	[39]
*TP53* mutation	In vitro	Ibrutinib; PrimaMet; CP-31398	No impact	The sensitivity to ibrutinib-induced cell death was similar in both *TP53* wild-type and *TP53*-mutant cell lines; treatment with CP-31398 and PrimaMet resulted in a significant decrease in viability in both Waldenström Macroglobulinemia (WM)-derived cell lines and primary WM cells, regardless of TP53 mutational status.	[32]
*TP53* mutation	Total (n = 49); Mutation (n = 24%)	Idelalisib; Obinutuzumab	No impact	No evidence was found for any predictive value of *TP53* mutation for response.	[36]
*TP53* mutation	In vitro	PRIMA-1Met	No impact	No significant changes in TP53 levels following PRIMA-1Met treatment were observed; selectively knocking down TP53 did not affect PRIMA-1Met-induced apoptosis in WM cells.	[46]
*TP53* silencing	In vitro; Mice	G-1	Impact	G-1 increased the protein expression of *TP53* and its targets—p21, *BAX*, *BAD*, and *PUMA*—in BCWM-1 cells and CD19^+^ cells from a WM patient; increased TP53 protein expression was also observed in tumors retrieved from a SCID/NOD mouse treated with G-1.	[45]

BAD: Bcl-2-associated death promoter. BAX: Bcl-2-associated X protein. NOD: Non-Obese Diabetic. OS: Overall Survival. p21: Cell Circle Inhibitor p21. PFS: Progression Free Survival. PUMA: P53 Upregulated Modulator of Apoptosis. Ref.: Reference. SCID: Severe Combined Immunodeficiency. TP53: Tumor Protein 53. WM: Waldenström Macroglobulinemia.

## Abbreviations

The following abbreviations are used in this manuscript:

ARID1A	AT-rich Interaction Domain 1A
ASPEN	A Study of Pharmacodynamics and Efficacy in Non-Hodgkin Lymphoma
BAD	Bcl-2-Associated Death promoter
BAX	Bcl-2-Associated X protein
BTK	Bruton’s Tyrosine Kinase
CBA	Chromosome Banding Analysis
CD19	Cluster of Differentiation 19
CD79A	Cluster of Differentiation 79A
CD79B	Cluster of Differentiation 79B
CHIP	Clonal Hematopoiesis of Indeterminate Potential
CXCR4	C-X-C Motif Chemokine Receptor 4
DLBCL	Diffuse Large B-Cell Lymphoma
DNA	Deoxyribonucleic Acid
FISH	Fluorescence In Situ Hybridization
GPER1	G-Protein-Coupled Estrogen Receptor 1
IGHV	Immunoglobulin Heavy Chain Variable
IgM	Immunoglobulin M
LPL	Lymphoplasmacytic Lymphoma
MDM2	Mouse Double Minute 2
MGUS	Monoclonal Gammopathy of Undetermined Significance
MWCL-1	Mantle-Cell Lymphoma Cell Line 1
MYD88	Myeloid Differentiation Primary Response 88
NF-κB	Nuclear Factor kappa-light-chain-enhancer of activated B cells
OS	Overall Survival
P21	Cell Circle Inhibitor P21
PFS	Progression-Free Survival
PUMA	p53 Upregulated Modulator of Apoptosis
TMNs	Therapy-related Myeloid Neoplasms
TP53	Tumor Protein 53
TTP	Time To Progression
TTT	Time To Treatment
WHIM	Warts, Hypogammaglobulinemia, Infections, and Myelokathexis
WM	Waldenström Macroglobulinemia
